# Implementation of a Physical Activity Vital Sign in Primary Care: Associations Between Physical Activity, Demographic Characteristics, and Chronic Disease Burden

**DOI:** 10.5888/pcd19.210457

**Published:** 2022-06-23

**Authors:** Cindy Y. Lin, Nicole L. Gentile, Levi Bale, Melanie Rice, E. Sally Lee, Lisa S. Ray, Marcia A. Ciol

**Affiliations:** 1 University of Washington Department of Rehabilitation Medicine, Seattle, Washington; 2 The Sports Institute at UW Medicine, Seattle, Washington; 3 University of Washington Department of Family Medicine, Seattle, Washington; 4 University of Washington School of Medicine, Seattle, Washington; 5 Population Health Analytics, UW Medicine, Seattle, Washington; 6 Information Technology Services, UW Medicine, Seattle, Washington

## Abstract

**Introduction:**

Physical activity is important to prevent and manage multiple chronic medical conditions. The objective of this study was to describe the implementation of a physical activity vital sign (PAVS) in a primary care setting and examine the association between physical activity with demographic characteristics and chronic disease burden.

**Methods:**

We extracted data from the electronic medical records of patients who had visits from July 2018 through January 2020 in a primary care clinic in which PAVS was implemented as part of the intake process. Data collected included self-reported physical activity, age, sex, body mass index, race, ethnicity, and a modified Charlson Comorbidity Index score indicating chronic disease burden. We classified PAVS into 3 categories of time spent in moderate to strenuous intensity physical activity: consistently inactive (0 min/wk), inconsistently active (<150 min/wk), and consistently active (≥150 min/wk). We used χ^2^ tests of independence to test for association between PAVS categories and all other variables.

**Results:**

During the study period, 13,704 visits, corresponding to 8,741 unique adult patients, had PAVS recorded. Overall, 18.1% of patients reported being consistently inactive, 48.3% inconsistently active, and 33.7% consistently active. All assessed demographic and clinical covariates were associated with PAVS classification (all *P* < .001). Larger percentages of consistent inactivity were reported for female, older, and underweight or obese patients. Larger percentages of consistent activity were reported for male, younger, and normal weight or overweight patients.

**Conclusion:**

Using PAVS as a screening tool in primary care enables physicians to understand the physical activity status of their patients and can be useful in identifying inactive patients who may benefit from physical activity counseling.

**Summary**

**What is already known on this topic?**

Routine clinical use of a physical activity vital sign (PAVS) can help clinicians identify patients who are insufficiently active.

**What is added by this report?**

In the primary care population assessed, 18.1% reported being consistently inactive, 48.3% inconsistently active, and 33.7% consistently active, based on US national physical activity aerobic guidelines. Women (vs men), patients aged ≥55 years (vs <55 y), and patients who had an underweight or obese body mass index (vs normal or overweight) were more likely to be consistently inactive.

**What are the implications for public health practice?**

Patients who reported being consistently inactive also reported a higher burden of chronic disease, indicating that PAVS can help identify patients who may benefit from physical activity counseling and prescription.

**MEDSCAPE CME**

In support of improving patient care, this activity has been planned and implemented by Medscape, LLC and *Preventing Chronic Disease*. Medscape, LLC is jointly accredited by the Accreditation Council for Continuing Medical Education (ACCME), the Accreditation Council for Pharmacy Education (ACPE), and the American Nurses Credentialing Center (ANCC), to provide continuing education for the healthcare team.

Medscape, LLC designates this Journal-based CME activity for a maximum of 1.00 AMA PRA Category 1 Credit(s)™. Physicians should claim only the credit commensurate with the extent of their participation in the activity.

Successful completion of this CME activity, which includes participation in the evaluation component, enables the participant to earn up to 1.0 MOC points in the American Board of Internal Medicine’s (ABIM) Maintenance of Certification (MOC) program. Participants will earn MOC points equivalent to the amount of CME credits claimed for the activity. It is the CME activity provider’s responsibility to submit participant completion information to ACCME for the purpose of granting ABIM MOC credit.

Release date: June 23, 2022; Expiration date: June 23, 2023

**Learning Objectives**

Upon completion of this activity, participants will be able to:

1. Distinguish the percentage of US adults who are not physically active from the current study by Lin and colleagues
2. Describe limitations of the Physical Activity Vital Sign (PAVS) assessment as a screening tool
3. Analyze variables associated with higher rates of physical inactivity

**EDITOR**

Ellen Taratus, MS

Senior Editor

Preventing Chronic Disease 

Atlanta, GA

**CME AUTHOR**

Charles P. Vega, MD

Health Sciences Clinical Professor of Family Medicine

University of California, Irvine School of Medicine

Irvine, California

Disclosure: Charles P. Vega, MD, has disclosed the following relevant financial relationships:

Served as an advisor or consultant for: GlaxoSmithKline; Johnson & Johnson Pharmaceutical Research & Development, L.L.C.

**AUTHORS**

Cindy Lin, MD

University of Washington

Department of Rehabilitation Medicine

The Sports Institute at the University of Washington

Seattle, Washington

Nicole L. Gentile, MD, PhD

University of Washington

Department of Family Medicine

Seattle, Washington

Levi Bale, BEd

University of Washington School of Medicine

Seattle, Washington

Melanie Rice, MA

The Sports Institute at the University of Washington

Seattle, Washington

E. Sally Lee, PhD

Population Health Analytics

University of Washington Medicine

Seattle, Washington

Lisa S. Ray, BA

Information Technology Services

University of Washington Medicine

Seattle, Washington

Marcia A. Ciol, PhD

University of Washington

Department of Rehabilitation Medicine

Seattle, Washington

## Introduction

Physical activity is central to a healthy lifestyle. In 2018, approximately one-quarter of US adults were physically inactive, reporting no leisure-time physical activity within the previous month (1), and approximately 20% were inconsistently active (2–4). The American Heart Association identified cardiorespiratory fitness as a predictor of mortality that is potentially stronger than risk factors such as smoking, hypertension, diabetes, or hyperlipidemia (5). Achieving the recommended 150 minutes per week of moderate-intensity aerobic activity improves cardiorespiratory fitness and can lower risk factors for many chronic diseases (2,4,5). Physical activity helps prevent and manage more than 40 conditions, including cardiovascular disease, obesity, cancer, depression, Alzheimer disease, and arthritis (2,6).

Several frameworks have been developed to promote physical activity in clinical practice, including the Exercise is Medicine (EIM) initiative. This framework comprises 3 components: physical activity assessment, brief counseling, and referral to resources (2). Health care delivery systems have used the framework to assess patients’ physical activity as a “vital sign,” similar to routine outpatient vital signs such as blood pressure (7,8). Brief physical activity assessment involves assessing patients’ frequency, intensity, time, and type of physical activity. The Physical Activity Vital Sign (PAVS) enables health care providers to identify patients who may benefit from physical activity counseling and prescription (2,6–8). PAVS is a tool with good face and discriminant validity when compared with national population-based surveys (8) and accelerometry data (9). Although primary care providers have variable levels of training and comfort in physical activity assessment and counseling (10), PAVS implementation during the clinic-rooming vitals-taking process (ie, the first face-to-face contact opportunity between clinical staff and patient during a visit) has been shown to be feasible (6,8).

Although physical activity can be assessed in clinical practice, few published studies have examined PAVS in the primary care setting and its relationship to chronic disease burden and demographic characteristics. Prior studies found that patients with greater disease burdens or who self-identified as racial or ethnic minorities tend to self-report lower rates of physical activity (8). Our primary study objective was to implement PAVS in a primary care clinic and to evaluate the association of PAVS with the demographic and clinical characteristics of patients.

## Methods

We conducted an observational study in which we assessed PAVS data from July 1, 2018, through January 31, 2020. The protocol was approved by the University of Washington Medical Center institutional review board. PAVS was implemented in a family medicine clinic in May 2018, and medical assistants asked PAVS questions during the rooming process of nonpregnant adult patients aged 21 years or older. The standardized PAVS questions were “How many days per week do you engage in moderate to strenuous exercise (like a brisk walk or breathing harder than normal)?” and “On average, each time you exercise, how many minutes do you exercise at this level?” (6) The initial PAVS implementation was in mid-May 2018 in 1 clinic pod, and it was expanded by July 2018 to the entire clinic. The start date for the study dataset was July 1, 2018, and the end date was January 31, 2020. We selected this end date because of the onset of the COVID-19 pandemic shortly thereafter, which affected staffing and outpatient clinic visits. We excluded visits without a recorded PAVS from the dataset and ascertained all unique patients in the remaining dataset. Patients reporting 0 minutes per week of physical activity consistently in all clinic visits were classified as consistently inactive, whereas patients reporting 150 minutes or more per week of physical activity in all visits were classified as consistently active. Patients who reported physical activity rates that did not fit the above 2 categories consistently for all visits were classified as inconsistently active (<150 min/wk).

We collected data from the health care system’s enterprise data warehouse, which included the electronic health records (EHRs) Epic encounters table with information on patient admission, discharge, and transfer. We assessed the following demographic characteristics: age; biological sex; race, categorized in the EHR as Asian, Black/African American, “Other” (American Indian/Alaska Native, Native Hawaiian/Pacific Islander, and multiple races), or White; ethnicity (Hispanic, non-Hispanic, and unknown); and body mass index (BMI). We assessed chronic disease burden by using a modified Charlson Comorbidity Index (CCI) (11,12), which was calculated by using *International Classification of Diseases, Tenth Revision, Clinical Modification* (ICD-10-CM) codes (13) for 17 health conditions from the 3 years before the first clinic visit with PAVS recorded. We included ICD-10-CM codes from all visits during that period. A higher CCI score is generally associated with increased age, comorbidities, and all-cause mortality (11,12).

### Statistical analysis

We conducted all statistical analyses in SPSS version 26 for Mac (IBM Corp). We tabulated data on demographic (sex, age, race, ethnicity) and clinical variables (BMI and modified CCI score) by PAVS group. We used the χ^2^ test of independence to assess the association of the demographic and clinical variables with each PAVS category. The significance level was .05; we made no adjustment for multiple comparisons because the study was exploratory.

Calculation of the modified CCI score for the study population was automated in a secure population health dashboard (HBI Solutions, Inc) that derives data from the Epic EHR. When no diagnostic codes included in the CCI definition were identified in any visits during the 3 years before the first recorded PAVS in the clinic, the algorithm listed a blank. Those blanks were transformed into CCI = 0. We additionally evaluated the age distribution by CCI group, which was consistent with the literature in which patients with 0 comorbidities or low CCI values (eg, 1 or 2) were younger and had fewer comorbidities (11,14).

## Results

From July 1, 2018, through January 31, 2020, a total of 43,939 visits were recorded for patients aged 21 years or older; of these visits, 30,235 visits (corresponding to 6,385 patients) did not have PAVS recorded. At the visit level, the overall PAVS capture rate was 31.2% (13,704 of 43,939 visits), which further corresponded to 8,741 unique patients (Figure).

**Figure:**
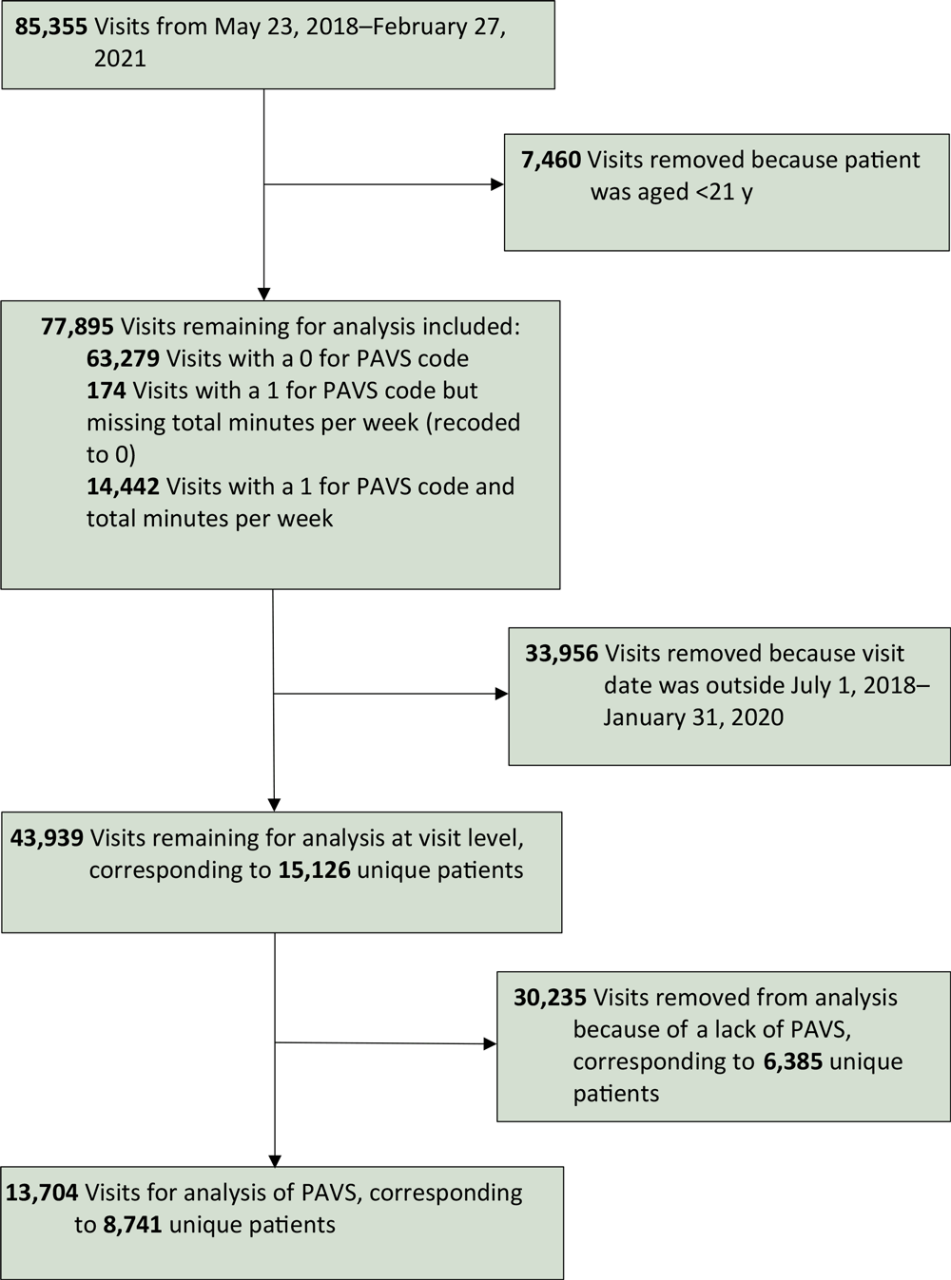
Patient selection for data analysis in a primary care clinic that explored the use of a physical activity vital sign (PAVS), United States, 2018–2020.

A little more than half (52.5%) of patients were women (n = 4,587); mean age was 45 years (SD, 17 y; range 21–104 y); and most were White (n = 5,239; 59.9%). Overall, 18.1% reported being consistently inactive and 33.7% consistently active (Table). The percentage of patients classified as consistently inactive or consistently active was greater among patients with a single visit (n = 5,745) than among patients with 2 or more visits (n = 2,996) (21.9% vs 10.7% and 40.3% vs 21.0%, respectively). This result was expected because a patient with more than 1 visit might report different levels of physical activity at different visits. All covariates were not independent of PAVS classification (all *P* < .001). The percentage of patients who were consistently inactive was larger among women (20.2%) than men (15.7%), among patients aged 55 or older (range, 20.8%–32.3%) than patients aged 54 years or younger (range, 14.5%–18.6%), among patients who were underweight (24.0%) or obese (21.9%) than patients who were normal weight (15.8%) or overweight (16.3%), and among patients with a modified CCI score of 3 or more. The percentage of patients who were consistently active was larger among men (38.4%) than women (29.4%), among patients aged 54 years or younger (range, 32.3%–37.3%) than patients aged 55 years or older (range 24.2%–31.3%), among patients who were normal weight (37.5%) or overweight (36.5%) than underweight (32.2%) or obese (26.1%).

**Table:** Distribution of Physical Activity Vital Sign (PAVS) Categories in a Sample of Patients in a Family Medicine Clinic, by Demographic Characteristics and Comorbidities, United States, 2018–2020^a^

Characteristic	No.	Level of activity,^b^ no. (%)			
		Consistently inactive	Inconsistently active	Consistently active	*P* value^c^
Entire sample of unique patients	8,741	1,578 (18.1)	4,218 (48.3)	2,945 (33.7)	—
Patients with a single visit	5,745	1,257 (21.9)	2,171 (37.8)	2,317 (40.3)	
Patients with ≥2 visits	2,996	321 (10.7)	2,047 (68.3)	628 (21.0)	
**Sex**					
Female	4,587	927 (20.2)	2,312 (50.4)	1,348 (29.4)	<.001
Male	4,154	651 (15.7)	1,906 (45.9)	1,597 (38.4)	
**Age group at first visit, y**					
20–34	3,186	463 (14.5)	1,534 (48.1)	1,189 (37.3)	<.001
35–44	1,585	260 (16.4)	788 (49.7)	537 (33.9)	
45–54	1,325	247 (18.6)	650 (49.1)	428 (32.3)	
55–64	1,206	251 (20.8)	577 (47.8)	378 (31.3)	
65–74	947	198 (20.9)	455 (48.0)	294 (31.0)	
≥75	492	159 (32.3)	214 (43.5)	119 (24.2)	
**BMI at first visit^d^**					
Underweight (<18.5)	146	35 (24.0)	64 (43.8)	47 (32.2)	<.001
Normal (18.5–24.9)	3,073	486 (15.8)	1,434 (46.7)	1,153 (37.5)	
Overweight (25.0–29.9)	2,784	455 (16.3)	1,312 (47.1)	1,017 (36.5)	
Obese (≥30.0)	2,535	555 (21.9)	1,319 (52.0)	661 (26.1)	
**Race**					
Asian	1,146	230 (20.1)	588 (51.3)	328 (28.6)	<.001
Black or African American	569	156 (27.4)	274 (48.2)	139 (24.4)	
Other^e^	227	54 (23.8)	112 (49.3)	61 (26.9)	
Unknown/declined to report	1,560	250 (16.0)	712 (45.6)	598 (38.3)	
White	5,239	888 (16.9)	2,532 (48.3)	1,819 (34.7)	
**Ethnicity**					
Not Hispanic or Latino	6,584	1,220 (18.5)	3,243 (49.3)	2,121 (32.2)	<.001
Hispanic or Latino	409	94 (23.0)	192 (46.9)	123 (30.1)	
Unknown/declined to report	1,748	264 (15.1)	783 (44.8)	701 (40.1)	
**Modified Charlson Comorbidity Index score^f^**					
0	6,929	1,137 (16.4)	3,290 (47.5)	2,502 (36.1)	<.001
1	902	193 (21.4)	487 (54.0)	222 (24.6)	
2	483	115 (23.8)	232 (48.0)	136 (28.2)	
3	161	50 (31.1)	76 (47.2)	35 (21.7)	
4	83	20 (24.1)	47 (56.6)	16 (19.3)	
≥5	183	63 (34.4)	86 (47.0)	34 (18.6)	

Abbreviations: —, does not apply; BMI, body mass index.

a Percentages across row may not add to 100 because of rounding.

b Patients reporting 0 minutes per week of physical activity consistently in all clinic visits were classified as consistently inactive; patients reporting ≥150 minutes per week of physical activity in all visits were classified as consistently active; patients who reported physical activity rates that did not fit the above 2 categories consistently for all visits were classified as inconsistently active (<150 min/wk).

c χ^2^ test for independence of variables.

d 203 (2.3%) patients were missing BMI values; percentages are based on the number of patients who had data on BMI. BMI calculated as weight in kg divided by height in meters squared.

e “Other” includes American Indian/Alaska Native, Native Hawaiian/Other Pacific Islander, and multiple races.

f Score was calculated by using the *International Classification of Diseases, Tenth Revision, Clinical Modification* codes (13) for 17 health conditions from the 3 years before the first clinic visit with PAVS recorded. A higher Charlson Comorbidity Index score is generally associated with increased age, comorbidities, and all-cause mortality (11–13).

By race, Black patients had the highest percentage (27.4%) reporting being consistently inactive, and White patients had the highest percentage (34.7%) reporting being consistently active. However, because 18% of patients declined to report their race, it is not possible to know in what direction the results would have been affected if their race had been reported. The same occurred for ethnicity: 20% (1,748 of 8,741) of patients did not declare their ethnicity in the EHR.

PAVS and modified CCI score were associated (Table): the percentage of patients who reported being consistently inactive increased as the modified CCI score increased, while the percentage of patients who reported being consistently active decreased as the modified CCI score increased.

## Discussion

In our study, 31% of all patient visits during the study period had a recorded PAVS. The PAVS capture rate in the clinic started at 15% to 20% during initial PAVS implementation, as clinic staff members were learning how to use it, and increased to as much as 43%. The percentage of PAVS recorded decreased in late 2019 after the clinic sustained a major flooding event, which disrupted clinical care. Soon after, in early 2020, the COVID-19 pandemic began and there were decreases in outpatient clinic visits.

Clinic staff members cited the following as barriers to implementing PAVS: lack of time, new or temporary staff members who were unfamiliar with PAVS, patients declining to state physical activity level or not knowing what their physical activity level was, a recent record of PAVS already existed, and unexpected circumstances affecting clinic workflow (ie, flooding and COVID-19 pandemic). Of the unique patient encounters with recorded PAVS, 34% of patients reported being consistently active and meeting the US national physical activity guidelines for aerobic activity, while 18% reported being consistently inactive. Regularly assessing a patient’s physical activity level is important in a clinical setting because this information might indicate a patient’s health status, as shown by the modified CCI findings, and help identify patients who may benefit from physical activity counseling. Prior studies found that physical activity and chronic disease burden are associated and that people who report higher levels of physical activity also have lower levels of chronic disease (15). Cause and effect could not be determined by our study because physical activity and chronic disease burden were measured cross-sectionally. However, our results show that certain patient groups may be more or less likely to be consistently active or inactive. Our findings suggest that in a similar primary care setting, female, older, and underweight or obese patients may benefit from targeted physical activity assessment and counseling.

Likely reasons for discrepancies in physical activity levels based on age and sex include self-identified barriers. Among middle-aged and elderly patients, self-identified barriers to exercise include cost, physical activity interference with family activities, time, lack of an exercise companion or facilities, fatigue, motivation, and current level of activity perceived as “enough” (16). Psychosocial factors (eg, self-efficacy, social support, motivation) are also cited as potential contributors to disparities in physical activity between men and women (17). Understanding these barriers is an integral step toward reducing physical activity disparities and tailoring effective interventions to increase physical activity among patients.

Lower levels of physical activity among people with high BMI could be the manifestation and a byproduct of physical activity barriers, including time constraints, limited access to facilities, fear of injury, and feelings of self-consciousness as described in the literature (17). The low physical activity level among people with a BMI of 30 or more may also be a consequence of the limitations related to higher disease burden, consequently presenting more barriers to consistent physical activity (18). Conversely, a subset of underweight patients, because of their health conditions or age, may also need additional support and resources to facilitate appropriate levels of physical activity.

An increased modified CCI score has been associated with an increased risk of all-cause mortality over time (19). In our study, patients with a higher modified CCI score more commonly reported consistent inactivity and less commonly reported consistent activity. From this cross-sectional study, it is impossible to assess whether high disease burden was a consequence of inactivity or if high disease burden precluded a person from being active.

Strengths of this study include a relatively large sample size, a broad age distribution for both men and women, and a study population diverse in disease pathology and race and ethnicity. Additionally, this study demonstrated a successful population-based approach to assessing physical activity levels among primary care patients. The approach used validated questions to identify patients that may benefit from physical activity counseling and prescription.

A limitation of this study is that data came from a single primary care clinic, and thus, the study may not be generalizable to clinics that differ in practice or in geographic location. Additionally, several limitations are inherent in EHR-derived data, including missing patient self-reported information. Although we found differences in PAVS across self-reported racial categories, the percentage of patients who selected “unknown/declined to answer” for race and ethnicity during their health care visit made it difficult to assess the clinical and statistical significance of these variables as they related to PAVS and the modified CCI score.

The modified CCI score was calculated according to all billed ICD-10-CM diagnoses coded in the EHR during the 3 years before the study period. If a patient’s diagnosis was recorded before the previous 3 years and not carried forward or if the patient was new to the health care system, the diagnosis may not have been entered into the EHR, and thus, this information could have been missing from the CCI calculation. However, this scenario is unlikely because the medical conditions included in the modified CCI tend to be discussed frequently during patient visits.

Several limitations to PAVS assessment have been reported. Psychosocial factors such as social desirability, social approval (20), and the degree of one’s exercise identity (21) can lead to variable accuracy in the self-reporting of physical activity. Additionally, although PAVS has demonstrated validity and high test–retest reliability in racially and ethnically diverse populations (22), intercultural differences in the interpretation of exercise terms such as “vigorous” and “moderate” may still lead to variations in PAVS (23).

Another limitation is the classification of patient physical activity levels. Because some patients had more than 1 visit during data collection, our classification was more precise for patients who were consistently inactive or consistently active than it was for patients who had some activity or were inconsistently active. A patient who had more than 1 visit might report being very active during a regular checkup but report no physical activity on a subsequent visit for an acute issue, which could result in a misleading classification of inconsistently active.

PAVS is a tool with face and discriminant validity in the rooming process (8). However, because of turnover in clinical staff and providers over the years, not every visit had a recorded PAVS. Selection bias could be improved by consistently assessing all patients for physical activity during all clinic visits, as is done for blood pressure and heart rate. Furthermore, collecting PAVS at every visit, including acute problem–based visits or follow-up visits, may affect the measurement of physical activity in the clinic population. Studies are needed to compare PAVS between annual checkups and other visit types, such as new patient visits and acute problem–based visits to analyze associations of PAVS and common ICD-10-CM codes.

Several key changes to future studies could affect data collection and allow us to improve our understanding of physical activity and its effects on disease burden. Future PAVS questionnaires should be validated in linguistically diverse settings to understand the appropriate terminology for describing physical activity intensity and types. To account for reporter bias in self-reported physical activity, some studies used accelerometers, pedometers, and smartwatches (20,24,25). These should be considered in future studies. Clinicians and researchers should work with the developers of EHRs to standardize and implement PAVS at all outpatient visits to provide more complete data across health care systems.

Using PAVS as a screening tool in primary care settings allows practicing physicians to rapidly identify the physical activity status of their patients. In this family medicine clinic, women, older patients, underweight or obese patients, and patients who had an elevated modified CCI score more often than their counterparts self-reported consistent inactivity or inconsistent activity. With this information, a provider can then provide brief physical activity counseling as recommended by the Exercise is Medicine initiative (1). Further research needs to be conducted to determine the correlation between PAVS and disease burden and evaluate whether routine clinical use of PAVS followed by physical activity counseling and referrals can lead to improved health outcomes in diverse patient populations.

## Post-Test Information

To obtain credit, you should first read the journal article. After reading the article, you should be able to answer the following, related, multiple-choice questions. To complete the questions (with a minimum 75% passing score) and earn continuing medical education (CME) credit, please go to http://www.medscape.org/journal/pcd. Credit cannot be obtained for tests completed on paper, although you may use the worksheet below to keep a record of your answers.

You must be a registered user on http://www.medscape.org. If you are not registered on http://www.medscape.org, please click on the “Register” link on the right hand side of the website.

Only one answer is correct for each question. Once you successfully answer all post-test questions, you will be able to view and/or print your certificate. For questions regarding this activity, contact the accredited provider, CME@medscape.net. For technical assistance, contact CME@medscape.net. American Medical Association’s Physician’s Recognition Award (AMA PRA) credits are accepted in the US as evidence of participation in CME activities. For further information on this award, please go to https://www.ama-assn.org. The AMA has determined that physicians not licensed in the US who participate in this CME activity are eligible for AMA PRA Category 1 Credits™. Through agreements that the AMA has made with agencies in some countries, AMA PRA credit may be acceptable as evidence of participation in CME activities. If you are not licensed in the US, please complete the questions online, print the AMA PRA CME credit certificate, and present it to your national medical association for review.

## Post-Test Questions

### Study Title: Implementation of a Physical Activity Vital Sign in Primary Care: Associations Between Physical Activity, Demographic Characteristics, and Chronic Disease Burden

#### CME Questions

1. What was the percentage of adults reporting no leisure-time PA in the current study by Lin and colleagues?
  - 18%
  - 37%
  - 51%
  - 68%
2. You are seeing a 45-year-old Asian woman with a history of type 2 diabetes for a 6 month follow up. You ask her to classify her current level of PA. Which of the following is not a limitation to the Physical Activity Vital Sign (PAVS) assessment?
  - Psychosocial factors
  - Use in racially/ethnically diverse populations
  - Intercultural differences in the interpretation of exercise terms
  - Classification of patient-reported PA levels
3. You ask the patient if she likes any type of PA. Which of the following variables was significantly associated with physical inactivity in the current study?
  - Male sex
  - Age 20 to 34 years
  - Obese but not underweight
  - Higher number of comorbid illnesses
4. Which of the following racial/ethnic groups had the highest rate of physical inactivity in the current study?
  - White
  - Black
  - Asian
  - Latino
